# Alzheimer’s disease: a mini-review for the clinician

**DOI:** 10.3389/fneur.2023.1178588

**Published:** 2023-06-22

**Authors:** Rishi S. Madnani

**Affiliations:** Bates College, Lewiston, ME, United States

**Keywords:** neurodegeneration, medicine, pathophysiology, history, Alzheimer’s disease, diagnosis

## Abstract

Alzheimer’s disease (AD), the most common form of dementia, is a striking example of the connection between neurophysiological abnormalities and higher-order cognitive deficiencies. Since its initial description in 1906, research into the pathophysiology and etiology of AD has led to the illumination of an incredibly complex set of genetic and molecular mechanisms for the disease’s progression, characterized by much more than the neuropathological hallmarks of beta-amyloid (Aβ) plaques and neurofibrillary tangles (NFTs). In this review, findings relating the neurodegeneration present in AD to its clinical presentation and treatment are summarized, with an emphasis on the interconnectedness of disease pathophysiology. Further, diagnostic guidelines are provided based on the National Institute on Aging-Alzheimer’s Association (NIA-AA) workgroup’s clinical recommendations. Through the dissemination of detailed but digestible open access resources such as this one, we can move towards an increase in the equity and accessibility of education for the modern clinician.

## Pathophysiology

Since its discovery and classification, the pathophysiology of Alzheimer’s disease has been studied extensively, leading to confirmation of the two prominent neuropathological motifs that Alois Alzheimer (1) and Oskar Fischer (2) first identified in the early 1900s: plaques and neurofibrillary tangles (3). The first, senile plaques, are defined as extracellular aggregates of beta-amyloid protein (Aβ), which is produced through proteolytic cleavage of a critical membrane glycoprotein, amyloid precursor protein (APP) (4). While APP can be differentially cleaved by β-secretase and γ-secretase to produce Aβ peptides of various lengths, it is the 42 amino acid form that is predominantly involved in plaque formation (5), as a result of its decreased solubility and increased propensity for fibril assembly. These fibrils, while largely implicated in Aβ plaque formation, represent only one of the potential polymeric forms of Aβ.

Because a variety of studies have shown that Aβ plaque formation is not correlated with the incidence or severity of Alzheimer’s (6), attention has largely turned to the oligomeric form of Aβ, which is soluble and capable of spreading throughout the brain *via* the cerebrospinal fluid (CSF) (7). These oligomers have the potential to bind to a number of extracellular receptors (8), at least one of which (PrP^C^) is demonstrated to recognize and bind Aβ fibrils and oligomers, preventing their elongation and contributing to the formation of short and highly neurotoxic Aβ polymers (9). Upon binding, their cytotoxic effects seem to be mediated by disrupted Ca^2+^ signaling, oxidative stress, and mitochondrial dysfunction (10). Notably, a 2002 paper published in *Nature* showed that Aβ oligomers, in the absence of monomers and fibrils, significantly inhibit long-term potentiation in the hippocampus of rats (11). Many studies have attempted to determine the mechanism for this neuronal damage; for example, a 2020 study found that incubation with soluble Aβ oligomers led to sensitization of Toll-like Receptor 4 (TLR4) and increased production of Tumor Necrosis Factor-ɑ (TNF-ɑ) in murine microglia and astrocytes (12). However, research into the role of Aβ in Alzheimer’s is far from complete—in the light of news that data from a critical 2006 study demonstrating the role of 56 kDa Aβ oligomers in murine impairment (13) was likely falsified, new findings are of critical importance in affirming the validity of existing research.

The second widely recognized component of Alzheimer’s pathophysiology is the presence of intracellular neurofibrillary tangles (NFTs) (4). The primary structural constituents of these tangles, paired helical filaments (PHFs), and single filaments (SFs), have a common structural origin and differ primarily in their aggregation pattern (14). These filaments result from the entanglement of abnormally hyperphosphorylated tau protein (15); as this protein plays a critical role in microtubule assembly and maintenance, its phosphorylation at certain Serine/Threonine residues alters its chemical and physical properties such that it can no longer fulfill its biological function (16). Specifically, it has been found that hyperphosphorylated tau protein is unable to bind tubulin (which is critical for its role in microtubule assembly), but readily binds to normal tau protein and other microtubule-associated proteins (17), contributing to the loss of cytoskeletal microtubules (18) and increased intracellular tau aggregation observed in the Alzheimer’s brain. Further, tau hyperphosphorylation contributes to intracellular tau mislocalization, including to the dendritic spine where it contributes to synaptic dysfunction (19).

**Figure 1:**
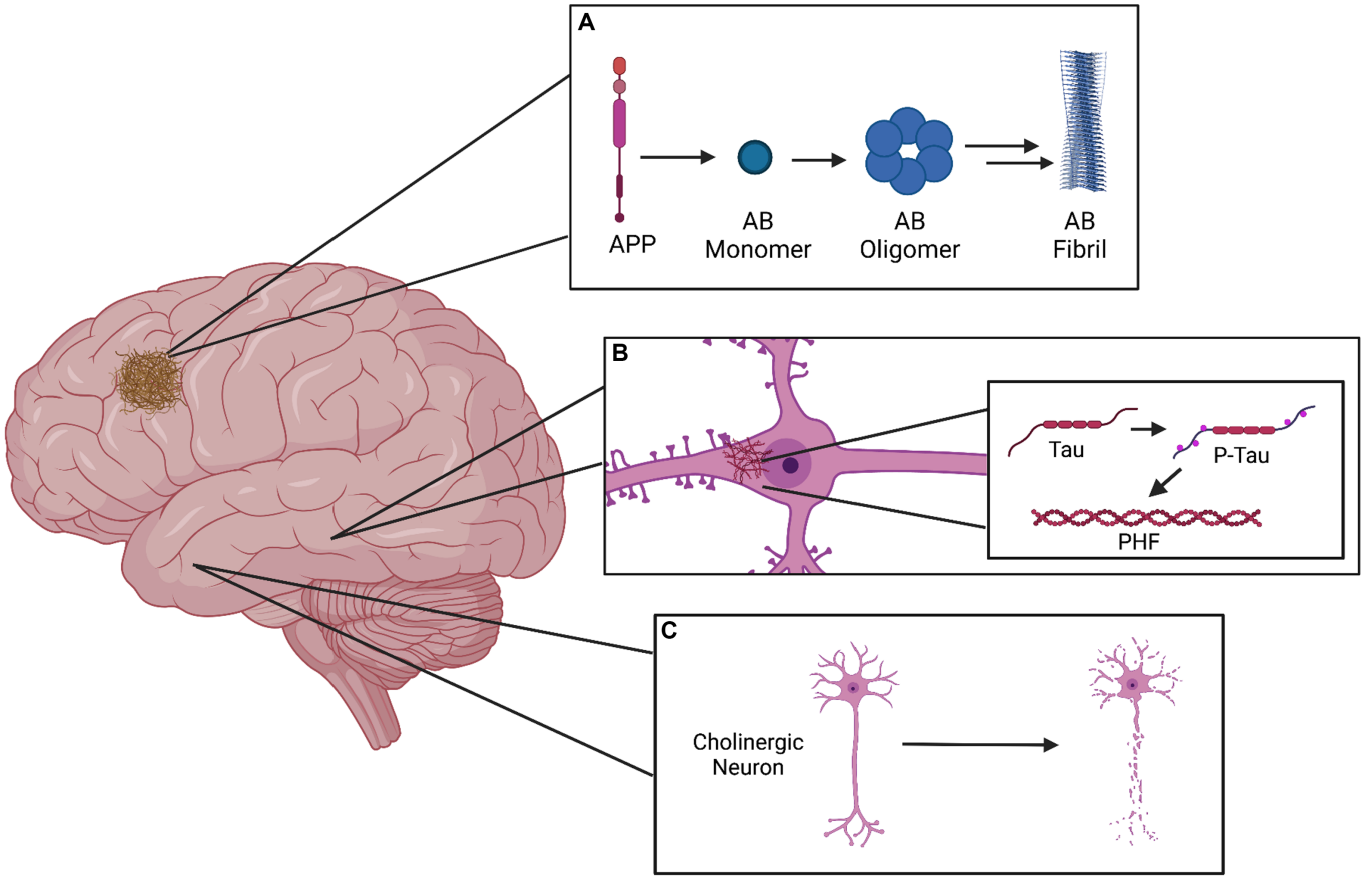
Graphical representation of the three primary pathophysiological manifestations of Alzheimer’s disease. **(A)** The amyloid plaque in the cerebral cortex is formed as a result of APP cleavage to form the Amyloid-beta monomer, which can subsequently form soluble oligomers. These oligomers can exert neurotoxic effects as they are, but may also assemble into insoluble protofibrils and later fibrils, which compose the plaques. **(B)** Depicted within a pyramidal neuron of the hippocampus, the neurofibrillary tangle (NFT) is localized to the cell body and results from the hyperphosphorylation of tau protein, which aggregates into paired helical filaments (PHFs). **(C)** Death of basal forebrain cholinergic neurons is a prominent marker of Alzheimer’s disease.

Further, the degeneration of cholinergic neurons in the nucleus basalis has been widely documented in the brains of patients with Alzheimer’s (20), eliciting an alternative, cholinergic hypothesis for the cognitive deficits observed in AD. However, research increasingly suggests that the various proposed mechanisms for Alzheimer’s pathophysiology may not be mutually exclusive; for example, exposure of cholinergic neurons to Aβ peptides has been shown to induce cytotoxicity, while activation of these neurons has also been shown to alter amyloid protein processing and tau protein phosphorylation (21). Further, Acetylcholinesterase (AchE) and presenilin 1 (the catalytic subunit of γ-secretase) have been shown to interact and modulate each other’s expression and activity (22). A simplified representation of the primary components of AD pathophysiology can be seen in Figure 1.

## Epidemiology and etiology

While the complex and multifaceted pathophysiology of Alzheimer’s continues to be studied, major attention has been given to the genetic, environmental, and social factors, which play a role in the development of this neurological degeneration. Primarily, aging is considered to be the dominant risk factor for the development of Alzheimer’s disease; only 1–6% of cases take the form of early-onset AD (EOAD), which is characterized by onset between the ages of 30–65 (23). Late-onset AD (LOAD) is much more common, with a current prevalence of around 6.5 million cases in the United States and a projected increase to 13.8 million by the year 2065 (23). Annual incidence of LOAD in the United States varies largely by age group, reflecting further the connection between aging and AD: in 2011, incidence was 0.4% in people age 65–74, 3.2% in age 75–84, and 7.6% in those above age 85 (23). Of the 6.5 million current cases, around 4 million are in women, while only 2.5 million are in men (23); however, the foundation for this difference is unclear, with a major factor simply being the increased life expectancy of women. Further, disparities in the incidence of Alzheimer’s exist across racial and ethnic lines, with Black and Hispanic Americans having a significantly higher rate of Alzheimer’s diagnosis than white Americans (23). The root of these disparities is difficult to determine, but evidence suggests that they are a result of the interaction between structural and biological factors that operate within the social construction of race (24). The cumulative effects of systemic racism on health cannot be understated (25); the impact of social and environmental factors such as exposure to pollutants/toxins, access to healthcare, and quality of education have led to a documented increase in many conditions which alter risk for AD, such as cardiovascular disease, diabetes, and depression (26).

Globally, more than 50 million people are estimated to be living with Alzheimer’s disease, and this number is projected to grow significantly (27); this increase is largely driven by population growth and an increase in average life expectancy, as well as improved methods of recognizing and diagnosing Alzheimer’s disease (specifically in less developed countries and impoverished communities within the United States).

Returning to the association of aging with Alzheimer’s disease, many general features of senescence (cognitive decline, metabolic deficiencies, and senile plaque/NFT formation) have the potential to contribute to the pathology of AD (28)—however, these factors also complicate the development of biomarker-based diagnostic tests, because many molecular patterns associated with AD pathology may not always be indicative of the condition, as is true of Aβ plaque formation (29).

By and large, the most explored risk factor for development of AD is the presence of certain deterministic or predisposing genes; in fact, studies show that the inheritability of AD is 50% or greater (30). Using genome-wide association studies, multiple such genes have been identified thus far, many of which have a direct connection to the aforementioned amyloid hypothesis. The first of these is *APP*, which encodes the amyloid precursor protein. As of 2020, there were 30 identified mutations in this gene, 25 of which lead to overproduction and accumulation of Aβ_42_ as a result of changes in the amino acid composition of APP’s cleavage site (4). Mutations in the genes *PSEN1* and *PSEN2*, whose protein products are involved in activation of the γ-secretase complex, are also considered to be causally related to AD development (31). The most prominent genetic risk factor, however, is the ε4 allele of the Apolipoprotein E (ApoE) gene, which is found in approximately 40% of people with AD (32). The ability of risk factors to modulate each other can be seen in the fact that DNA from HSV-1, infection with which is an established risk factor for AD, has been identified within the brains of those carrying the ApoE-ε4 allele (33).

Other established factors which alter the risk of developing AD include intelligence and educational attainment; while some suggest that these qualities may actually decrease the incidence of neural damage associated with AD, most advocate a framework (the cognitive reserve hypothesis) in which extensive early brain development allows one to maintain cognitive function in the presence of this damage (34). Beyond these factors, a variety of lifestyle-associated elements such as diet, physical activity, history of brain injury, and cardiovascular health have been statistically correlated with late-onset dementia (4), but the connection between these factors and specific Alzheimer’s pathology is inconclusive and requires further study. However, convincing research has been conducted on the role of stress in the development of AD pathology, specifically in regard to the role of corticotropin-releasing hormone signaling on Aβ and tau deposition, as well as neurodegeneration (35).

## Disease progression and presentation

Though various scales are used to evaluate the symptomatic and pathological progression of Alzheimer’s, an effective framework has been created by the National Institute on Aging and the Alzheimer’s Association (NIA-AA) to contain the three following stages: the preclinical, mild cognitive impairment (MCI), and dementia stages (36). The preclinical stage is characterized by the absence of AD signs/symptoms, but the presence of one or more biomarkers indicating the initiation of neuropathological patterns of AD (37). Within this stage, the NIA-AA working group has established a research framework for three substages characterized by the sequential accumulation of amyloidosis, neurodegeneration, and subtle cognitive decline (38); however, this framework is not yet recommended for implementation in clinical practice. Following this, early AD, often referred to as MCI, is accompanied by cognitive impairments in memory, executive function, language, attention, or visuospatial skills, but a preservation of functional ability and social/occupational functioning (39). To increase confidence in diagnosis of MCI specifically due to AD, the following criteria can be utilized: impairment of memory specifically, gradual decline of cognitive function (over months to years), lack of Parkinsonism and hallucination, lack of vascular/cerebrovascular risk factors, and lack of behavioral/language disorders (39). Once AD reaches the dementia stage, it can be characterized as moderate or severe. Moderate AD results in difficulty recognizing family/friends, greater memory loss, and significant changes in behavior, whereas severe AD eventually leads to a loss of communication and voluntary functions, including walking, urination, swallowing, and more; at this point in the disease, fatal complications are common and full-time care is recommended. The prognosis for Alzheimer’s varies depending on the time of diagnosis, but life expectancy at diagnosis is on average between 3 and 10 years (40), with progressive neurodegeneration expected to occur until death.

## Diagnosis

Diagnostically, the clinician is best suited to follow the criteria outlined by the National Institute on Aging and the Alzheimer’s Association (41). In order to be considered for the diagnosis of Alzheimer’s dementia, the patient must first meet the criteria for all-cause dementia, which state that the patient must exhibit neuropsychiatric symptoms which interfere with their occupational/social functioning, represent a decline from their previous state, are not explained by delirium or another mental disorder, display evidence of cognitive impairment (through medical history from the patient and an informant and the use of an objective cognitive assessment), and include impairment in two or more of the following domains: information acquisition and recall, reasoning and judgment in complex tasks, visuospatial ability, language function, and normal personality/behavior.

Following this diagnosis, AD status can be ascribed as follows: probable AD dementia, possible AD dementia, or probable/possible AD dementia with evidence of AD pathophysiology (41). For diagnosis of probable AD dementia, the following characteristics should be present: insidious onset, history of cognitive decline, and existence of most prominent deficits in one of the following domains: memory (most common), language, visuospatial, or executive function. Certainty in this diagnosis can be enhanced with documented decline of cognitive function and/or evidence of a causative genetic mutation in *APP*, *PSEN1*, or *PSEN2*. However, this diagnosis is not appropriate if the patient displays a history of concomitant cerebrovascular disease, features of Lewy body dementia (aside from the dementia), features of behavioral frontotemporal dementia, features of semantic variant primary progressive aphasia or nonfluent/agrammatic variant primary progressive aphasia, or evidence of a neurological or non-neurological disease or medication that may affect cognition—if any of these mixed etiologies are present, the patient should be diagnosed with possible AD. Similarly, a patient with atypical course (sudden onset of cognitive decline or insufficient demonstration of its progressive nature) should be diagnosed with possible AD.

Regardless of meeting the clinical criteria for possible or probable AD, evidence for a diagnosis of HIV dementia, dementia of Huntington’s disease, or another disease that rarely overlaps with AD renders a patient unlikely to have AD (41). If biomarker testing is utilized, negative Aβ and neuronal injury results also designate a patient as unlikely to have AD.

**Figure 2:**
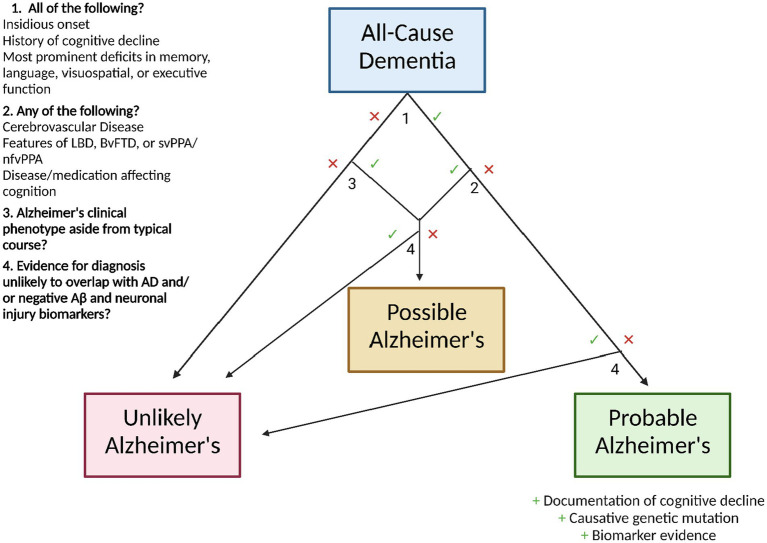
Workflow for Alzheimer’s disease diagnosis following All-Cause Dementia diagnosis, using the clinical guidelines of the National Institute on Aging and the Alzheimer’s Association.

The NIA-AA recommendations state that AD biomarker evidence can serve to increase certainty that the patient’s dementia is due specifically to the neurodegenerative pathophysiology of AD. The endorsed biomarkers, chosen based on their level of investigation and predictive accuracy (42), are as follows: low CSF Aβ_42_ and positive PET amyloid imaging for the indication of brain β-amyloid deposition, and elevated CSF tau (total and phosphorylated), decreased fluorodeoxyglucose uptake in PET of the temporoparietal cortex, and MRI-evidenced atrophy of the medial, basal, and lateral temporal lobes as well as the medial parietal cortex, and MRI-evidenced atrophy of the medial, basal, and lateral temporal lobes as well as the medial parietal cortex for the indication of neuronal degeneration. However, the working group does not yet advocate the routine integration of these biomarker-based tests into clinical practice due to the high accuracy of clinical criteria, ongoing investigation of biomarker predictive power, limited standardization of results, and inequitable distribution of testing technologies. It is important to note that these guidelines are those recommended by the NIA-AA; in 2021, the International Working Group released a set of recommendations for the diagnosis of Alzheimer’s that advocates an explicitly clinical-biological method, requiring biomarker evidence in the diagnosis of AD (43). The two sets of guidelines largely overlap aside from this point. A graphic workflow for the diagnosis of AD based on NIA-AA guidelines is shown in Figure 2.

## Treatment

While there is no cure for AD, there are a variety of treatment options which can modulate disease progression and improve quality of life for patients. Maintenance of cardiovascular fitness (44), continued intellectual activity (45), and consumption of plant-based foods (46) can all serve to strengthen cognitive function and decrease risk for AD, but ultimately, genetic predisposition to AD is considered to be of greater influence in the development of the condition (4).

Pharmacologically, AD symptoms can be reduced through the administration of two approved classes of drugs: acetylcholinesterase inhibitors, which inhibit the enzymatic breakdown of acetylcholine (47), and *N*-methyl D-aspartate (NMDA) receptor antagonists, which prevent the binding of glutamate to NMDA receptors in order to prevent cell death from neuronal overactivation (48). Approved AchE inhibitors include donepezil, galantamine, and rivastigmine, while the only approved NMDA receptor antagonist is memantine.

However, these drugs cannot cure or prevent Alzheimer’s disease; therefore, there is an increasing interest in the discovery of disease-modifying therapies, which function by inhibiting or slowing the pathophysiological development of AD (49). Many of these are monoclonal antibodies targeting Aβ—two of these therapies, Aducanumab and Lecanemab, have gained FDA approval for the treatment of AD and early AD, respectively. In clinical trials, both of these treatments led to moderate decreases in the rate of cognitive impairment in AD patients (50, 51), with respective differences of −0.39 and − 0.45 on change in Clinical Dementia Rating Scale Sum of Boxes (CDR-SB) score compared to placebo. However, there is a great deal of controversy regarding the approval of these drugs, considering their modest (and potentially clinically insignificant) effect on cognitive decline and increased risk for neurological complications (52).

## Links to inflammation and T2DM

Alzheimer’s is often comorbid with a number of health conditions, the most common of which are hypertension, diabetes, depression, osteoarthritis, and cerebrovascular disease (53). By examining the molecular patterns present in both AD and these conditions, a great deal of insight into the disease can be derived—for example, many of these conditions entail a significant inflammatory response. Research on the link between neuroinflammation and Alzheimer’s pathology has yielded promising results, demonstrating that microglial activation and cytokine (TNF-𝛼, IL-1β, IL-6, NFkB, IL-10, and TGF-β1) release within the brain are not only reactions to core AD pathologies, but also contributing factors to their progression (54).

Considering the critical role of inflammation in the development of insulin resistance and Type 2 Diabetes Mellitus (T2DM) (55), a body of research investigating the role of insulin dysregulation in AD has emerged. In the central nervous system, insulin has important functions including glucose homeostasis, neuronal growth and survival, and neurotransmitter synthesis (56). Brain hyperinsulinemia and insulin resistance has been shown to increase Aβ accumulation (57), demonstrating a link between the two conditions. A number of other findings have demonstrated causal relationships between insulin resistance and the development of AD pathology, prompting researchers to coin the term “Type 3 Diabetes” to acknowledge this link (58). In confirmation of this hypothesis, the ApoE ε4 protein was shown to impair CNS insulin signaling in mice by trapping insulin receptor in the endosomes; this effect was exacerbated when the mice were given a high-fat diet (59). Research in this field is ongoing, but findings thus far suggest that both pharmacological and lifestyle-based treatment of AD should include management of insulin resistance.

## Conclusion

As global life expectancy continues to increase, so will AD prevalence; thus, it is of critical importance that the scientific community continues to explore this condition and devotes efforts toward the development of novel therapeutics, which may inhibit AD pathology and neurodegeneration. For the clinician, adherence to established diagnostic guidelines, acknowledgement of the interconnectedness of AD pathology, and awareness of novel treatment options are critical for ensuring proper patient care.

## Author contributions

The author confirms being the sole contributor of this work and has approved it for publication.

## Funding

The publication of this work was supported by the Dean of the Faculty’s Office at Bates College.

## Conflict of interest

The author declares that the research was conducted in the absence of any commercial or financial relationships that could be construed as a potential conflict of interest.

## Publisher’s note

All claims expressed in this article are solely those of the authors and do not necessarily represent those of their affiliated organizations, or those of the publisher, the editors and the reviewers. Any product that may be evaluated in this article, or claim that may be made by its manufacturer, is not guaranteed or endorsed by the publisher.
